# Child murder in the Early Bronze Age: proteomic sex identification of a cold case from Schleinbach, Austria

**DOI:** 10.1007/s12520-020-01199-8

**Published:** 2020-10-23

**Authors:** Katharina Rebay-Salisbury, Lukas Janker, Doris Pany-Kucera, Dina Schuster, Michaela Spannagl-Steiner, Lukas Waltenberger, Roderick B. Salisbury, Fabian Kanz

**Affiliations:** 1 Institute for Oriental and European Archaeology, Austrian Academy of Sciences, Hollandstraße 11–13, 1020 Vienna, Austria; 2 Department of Analytical Chemistry, University of Vienna, Währinger Straße 38, 1090 Vienna, Austria; 3 Department of Anthropology, Natural History Museum, Burgring 7, 1010 Vienna, Austria; 4 Institute of Prehistoric and Historical Archaeology, University of Vienna, Franz-Klein-Gasse 1, 1190 Vienna, Austria; 5 Center for Forensic Medicine, Medical University of Vienna, Sensengasse 2, 1090 Vienna, Austria

**Keywords:** Bronze Age, Infanticide, Impression fractures, Otitis, Proteomic sex identification, Peptides

## Abstract

The identification of sex-specific peptides in human tooth enamel by nanoflow liquid chromatography-tandem mass spectrometry (nanoLC-MS/MS) represents a quantum leap for the study of childhood and social relations more generally. Determining sex-related differences in prehistoric child rearing and mortality has been hampered by the insufficient accuracy in determining the biological sex of juveniles. We conducted mass spectrometric analysis to identify sex-specific peptides in the dental enamel of a child from a settlement pit of the Early Bronze Age settlement of Schleinbach, Austria (c. 1950–1850 bc). Four perimortal impression fractures on the skull of a 5–6-year-old child indicate an intentional killing, with a co-buried loom weight as possible murder weapon. Proteomic analysis, conducted for the first time on prehistoric teeth in Austria, determined the child’s sex as male. While we cannot conclusively determine whether the child was the victim of conflicts between village groups or was slain by members of his own community, we suggest that contextual evidence points to the latter. A possible trigger of violence was the follow-on effects of an uncontrolled middle ear infection revealed by an osteological analysis. The boy from Schleinbach highlights the potential for further investigation of gender-biased violence, infanticide and child murder based on the recently developed method of proteomic sex identification.

**Electronic supplementary material:**

The online version of this article (10.1007/s12520-020-01199-8) contains supplementary material, which is available to authorized users.

## Introduction

This article brings together multiple strands of analysis to investigate the killing of a 5–6-year-old child from Early Bronze Age Schleinbach, Austria (c. 1950–1850 bc). Traces of interpersonal violence are common in this period and have given rise to numerous investigations of weaponry, defensive architecture and trauma on human remains (e.g. Osgood and Monks 2000; Otto et al. 2006; Parker Pearson and Thorpe 2005; Peter-Röcher 2007; Uckelmann and Mödlinger 2011). Bronze Age warfare in Europe has been described as an inter-group raiding of war-bands (Harding 2007), which contributed to the warrior’s honour and prestige and the shaping of male identity (Frieman et al. 2017; Treherne 1995), but it also included ritual and religious connotations (Kristiansen and Larsson 2005).

Archaeologists increasingly recognise that Bronze Age warfare was not only confined to conflicts between warriors but also included other individuals, such as women and children. At Herzogbierbaum, Austria, for example, a recent excavation revealed seven young women and children in a pit; four of them showed perimortal traumata (Lauermann and Pany-Kucera 2012). Capture and enslavement (Cameron 2013, 2016), particularly of women and children, may have played a large role in Bronze Age mobility. Moreover, Bronze Age cemeteries often do not include the expected numbers of infants and children (Beilke-Vogt 2004; Storch 2002).

Despite this body of evidence, it is rare that we can identify causes for violence and reconstruct a sequence of events. Furthermore, when the victims are children, only in exceptional cases can we determine the sex of the victim. The difficulty of sexing children via morphological traits has provided an obstacle to the interpretation of infanticide, filicide and gender-biased violence against children.

Tooth enamel contains sex chromosome-linked isoforms of amelogenin, an enamel forming protein, which preserve well even in archaeological and palaeontological specimens (Cappellini et al. 2019; Hendy et al. 2018). The identification of sex-specific peptides in human tooth enamel by nanoflow liquid chromatography-tandem mass spectrometry (nanoLC-MS/MS) represents a quantum leap for the study of childhood and social relations more generally. Recent studies have shown promising analysis strategies for sex identification based on proteome wide screening or individual diagnostic peptides (Parker 2019; Stewart et al. 2016, 2017). Furthermore, it has been demonstrated that the reliability of proteomic sex determination is improved over other strategies including shotgun genomics and osteological estimation (Buonasera et al. 2020).

Here, we are able to determine the victim’s sex by combining multiple proteomic data interpretation strategies, identify a potential trigger for violence, reconstruct the sequence of actions resulting in four perimortal impression fractures of the skull and suggest a possible murder weapon, all in one archaeological cold case. The detailed evidence from Schleinbach serves as a case study to discuss the context of violence against children in Early Bronze Age Central Europe.

## Study Area

### Early Bronze Age Schleinbach

The site Schleinbach is located near Vienna in Lower Austria and encompasses settlement traces as well as 36 graves and nine storage pits that included the remains of 64 individuals, which have been documented in the course of clay extraction for a brick factory that started in 1911 (Rettenbacher 2004). The site is part of the Early Bronze Age Únětice Culture complex, distributed from Thuringia and Saxony over Bohemia and Moravia to Silesia, Slovakia and Lower Austria (von Schnurbein 2009). Radiocarbon dates from six individuals from Schleinbach span 2084–1627 cal bc, with the most likely occupation of the site dating to c. 1950–1850 bc.

Life at Early Bronze Age Schleinbach took place in a small village of a few houses and included crops and keeping animals such as cattle, sheep/goat and pigs. Pottery, stone tool and textile production are well documented, and some bronze jewellery was in circulation, such as neck rings, dress pins and twisted wire rings to decorate hair or wear around the neck (Rettenbacher 2004).

Bronze grave goods and the way people were buried appear to have been used as markers of social distinction. Men and women were usually placed in graves in flexed position on the right side of the body, but a considerable number were buried in decommissioned storage pits. Typical Early Bronze Age storage pits are circular in plan and have a narrower top than bottom, making them appear trapezoid to pear-shaped in profile. The shape is functional and stable, providing a secure place to store grain. The placement of bodies within such pits varies; some follow body placement patterns used for graves, some appear haphazardly deposited, and others were secondary deposits in semi- or disarticulated position after primary burial elsewhere (Lauermann 2003; Neugebauer 1994).

A recent analysis of the human remains in the framework of the ERC-funded project ‘The value of mothers to society’ identified a high occurrence of traces of interpersonal violence, including healed and perimortal fractures (Pany-Kucera et al. 2020). For example, two maternally related adult males with identical impression fractures were buried in close bodily contact in one grave, and an adult man was deposited in a large pit together with three children aged 3–4, 8–9 and 12 years (Weninger 1954a, b). Bodies recovered from pits are rarely found with grave goods other than fragmented pottery and settlement refuse, and the health status of the buried individuals is often poor.

## Materials

### The storage pit

**Fig. 1:**
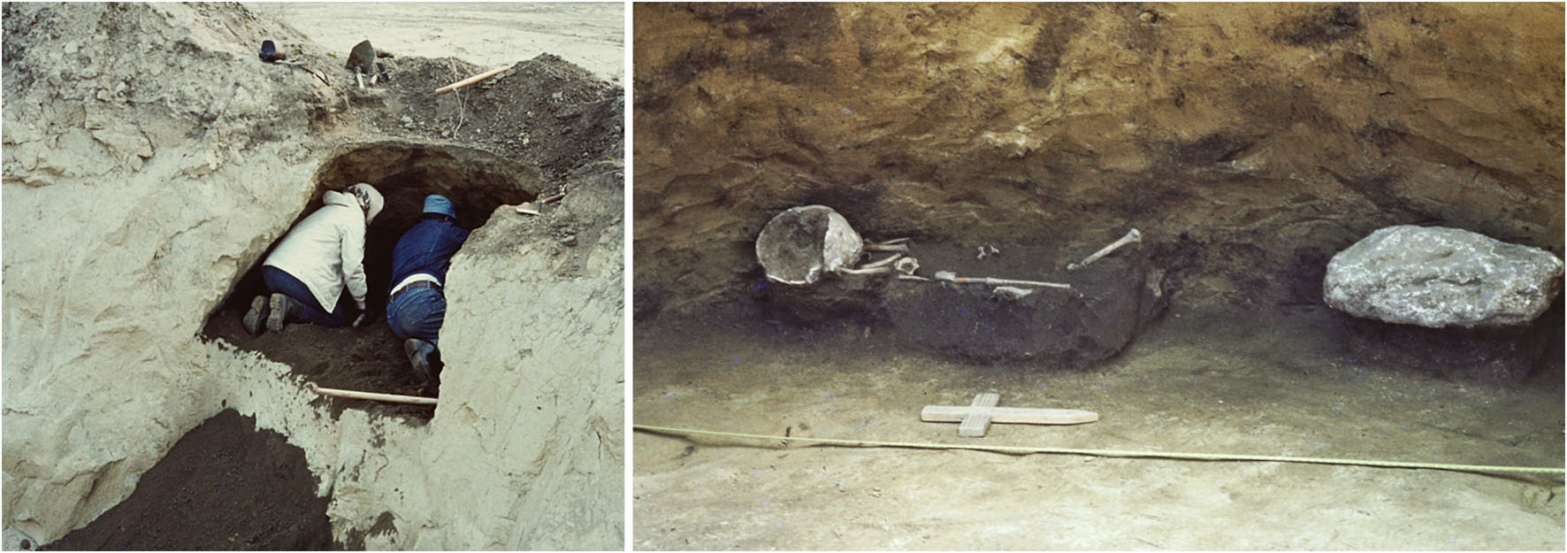
Early Bronze Age cylindrical pit (Grube 3) with the remains of a 5–6-year-old child excavated in 1981 (photos: H. Schwammenhöfer)

**Fig. 2:**
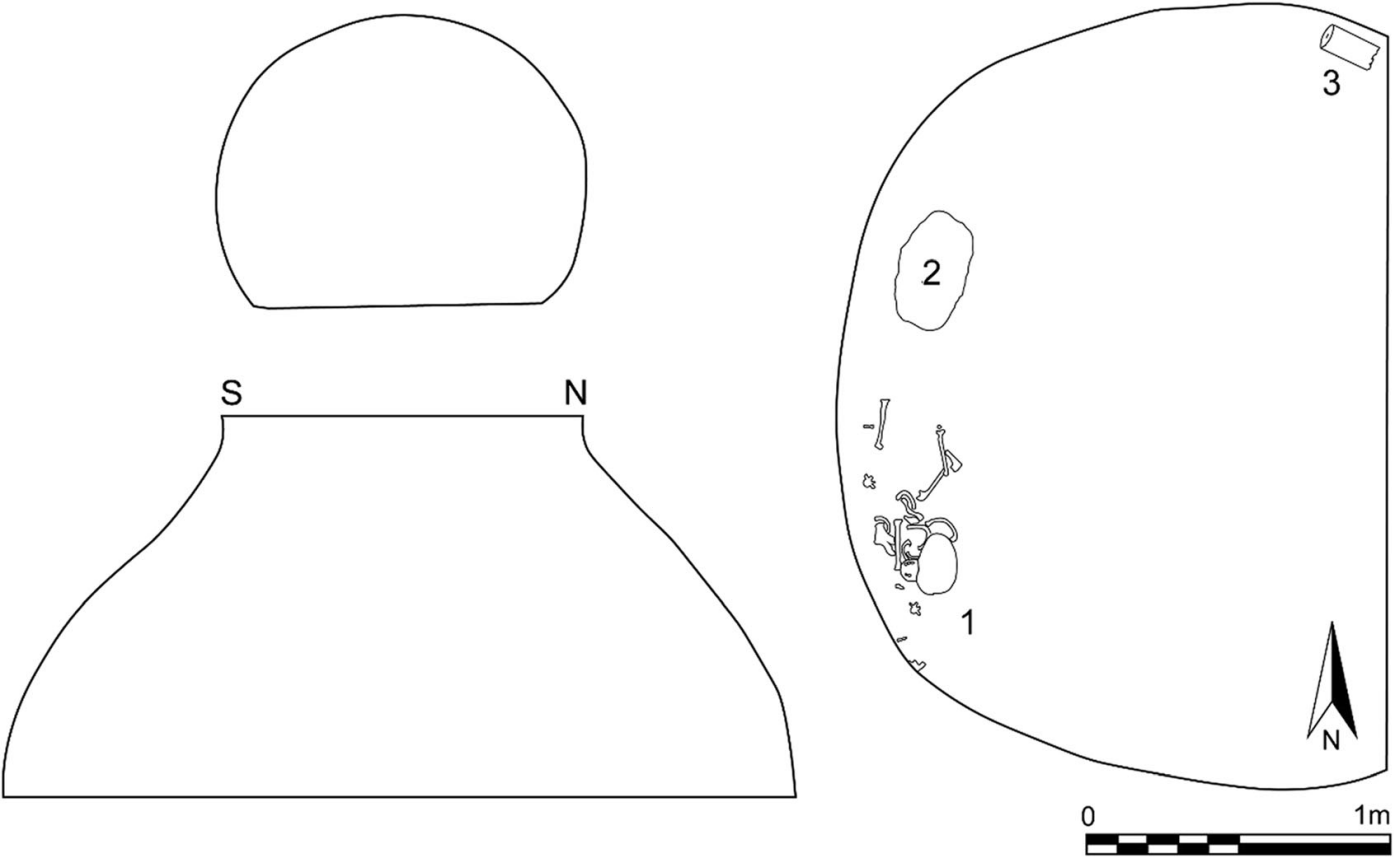
West profile and plan of the bottom surface of Pit 1981/Grube 3. 1, Remains of a 5–6-year-old child; 2, stone; 3, cylindrical loom weight

Human remains continued to come to light during clay extraction in the 1980s (Figs. 1 and 2). Hermann Schwammenhöfer came to the rescue of Pit 3 in 1981, when a quarter of the Early Bronze Age feature had already been removed. The top of the pit was 1.2 m in diameter, the bottom of the pit measured 2.6 m in diameter at a depth of 1.25 m underneath the top soil. It was filled with dark humus soil with some sherds and animal bones. Remains of a 5–6-year-old child were found deposited on a layer of dark soil about 0.1 m above the bottom, along the western wall of the pit (Schwammenhöfer 1981; Schwammenhöfer 1983).

The skeleton of the child was neither complete nor fully articulated; the remaining bones of the upper extremity, however, were found with the skull. It is unclear whether the body had decomposed in a different place in the open or in a grave first, and parts later moved into the pit, or whether this is the primary place of burial that was later disturbed. Preserved bones include the cranium and mandible, the left scapula and clavicle, some ribs and vertebrae, both humeri and the right radius and ulna. The skull was found turned with the face towards the wall and bottom of the pit. Traces of gnawing from the tiny teeth of mice were discovered at the margins of the depression fracture at the right parietal bone, the right orbital rim, the foramen magnum and at the crista frontalis at the inside of the frontal bone. This suggests that rodents gnawed through the soft tissue or had access to the skull at a time when the soft tissues had largely decomposed. The location of the gnawing marks could point to the pit having been left open for a while after the body (parts) have been deposited. Over time, the pit became completely filled.

**Fig. 3:**
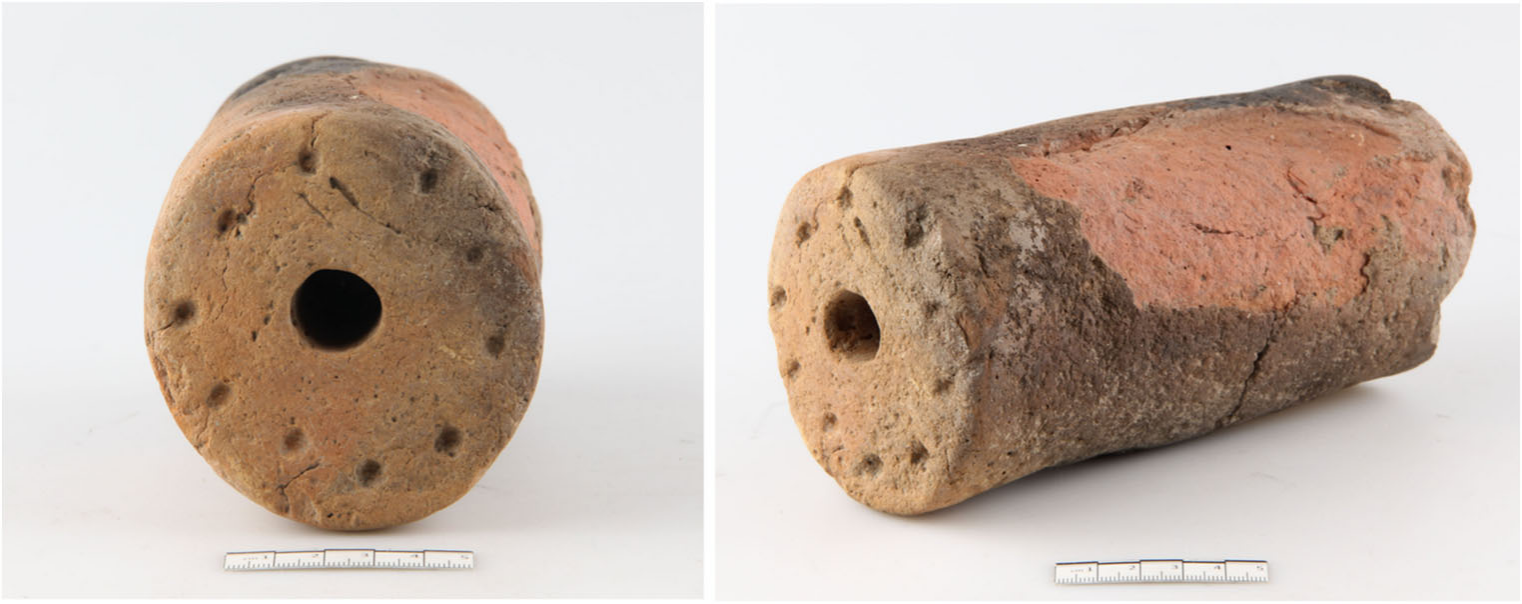
Loom weight found in of Pit 1981/Grube 3 (photo: Norbert Weigl, © Landessammlungen Niederösterreich)

A cylindrical loom weight was found in the north of the pit (Schwammenhöfer 1981), at the same levels as the child about 0.1 m above the bottom of the pit. It is 175 mm long, with a dimeter of 89 mm and a hole of 19 mm diameter through the entire length and weighs 1.505 kg (Fig. 3). The well-preserved base is decorated with 10 finger impressions around the central hole; the other side is worn and blackened from fire. The cylindrical shape of the loom weight is typical for the Early Bronze Age Únětice cultural complex and serves as an additional proxy for dating. Direct radiocarbon dating of the skeleton was impeded by post-excavation coating of the bone with clear household varnish. Two loom weights very similar in shape and decoration come from the settlement of Schleinbach itself (Rettenbacher 2004), and although loom weights are not typical grave goods, they are frequently found with burials in former storage pits, e.g. at Kleinhadersdorf (Lauermann 2003), Wetzleinsdorf (Kriegler 1925), Fels am Wagram (Engelhardt 1973) and Niederröblingen, Germany (Hubensack 2018).

A large stone was found between the child and the loom weight, again at the same level 0.1 m above the bottom of the pit, but was not recovered. Foreign to the loess soil of the site, the upper surface appears unworked, but the lower surface is flat; stones like these are used as grinding stones and are frequently found in Early Bronze Age domestic contexts. The fill of the pit further included a number of sherds, which can be interpreted as settlement refuse.

### The child from the pit

The child’s age was estimated at 5–6 years at death because of its full primary dentition and the lengths of the upper limb bones. The diaphyseal measurements of the right humerus (157–160 mm) and the right radius (127 mm) correspond to a body height of 1–1.05 m and an age of 4–6 years, with comparative data derived from radiographs of modern populations (Cunningham et al. 2016; Schmid and Künle 1958). In comparison with modern growth charts, the child would have been small for his age.

**Fig. 4:**
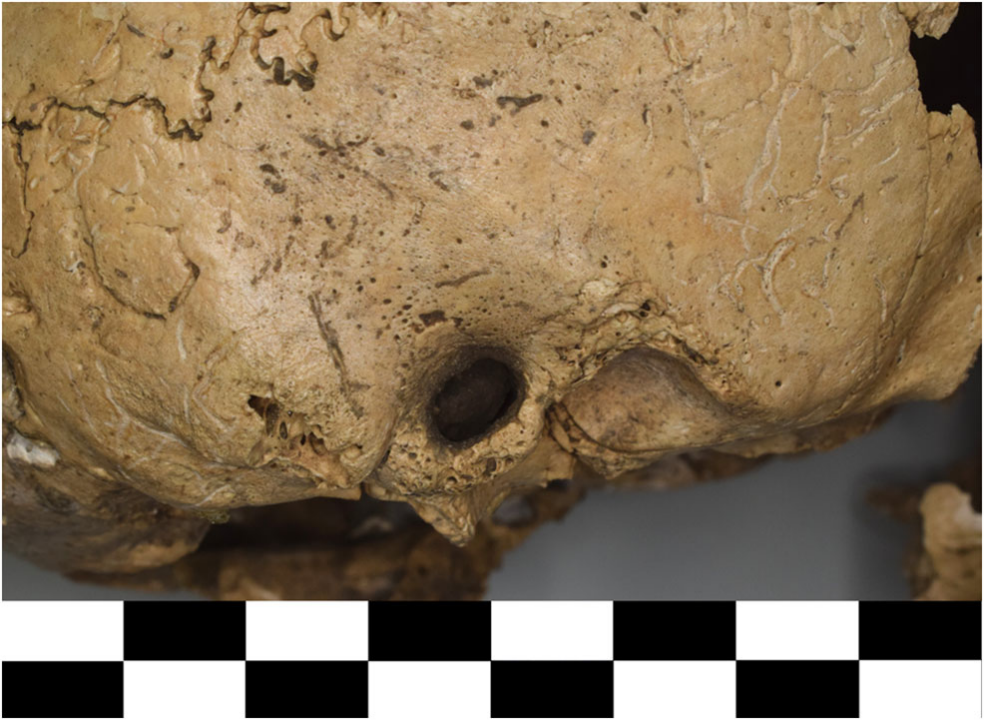
Mastoid process and auditory meatus of the 5–6-year-old child from Schleinbach

Intensified pitting and new bone formation were found at the maxilla, zygomatic bone, squamous part of temporal bone and the auditory meatus, indicating unspecific reactions to stress and/or deficiency symptoms. Porotic thickening at and around the external and probably internal auditory meatus on both sides might indicate otitis media, a complex bacterial infection causing inflammatory changes of the middle ear. The development of the mastoid process is usually also altered in otitis media, but this region is damaged on this specimen (Fig. 4). The anatomical variation of a persisting Foramen Huschke, albeit common at this age (Humphrey and Scheuer 2006), may have contributed to the spread of the infection. Otitis media, in acute or chronic form, is one of the most common infectious diseases affecting primarily children under seven. They were painful, dangerous and potentially lethal due to consecutive illnesses like meningitis, especially under prehistoric conditions, as no effective treatment was available (Krenz-Niedbała and Łukasik 2017). Hearing impairment might develop with the chronic form, which negatively influences the development of speech and children’s behaviour (Aarhus et al. 2015; Khairi et al. 2010).

**Fig. 5:**
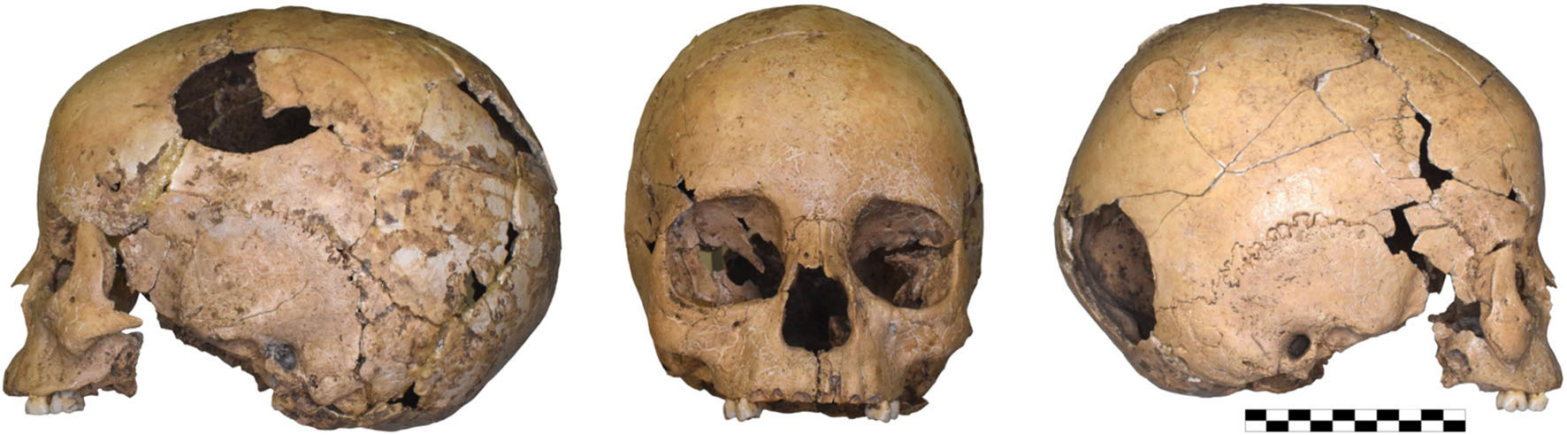
Cranium of the 5–6-year-old child from Schleinbach

**Fig. 6:**
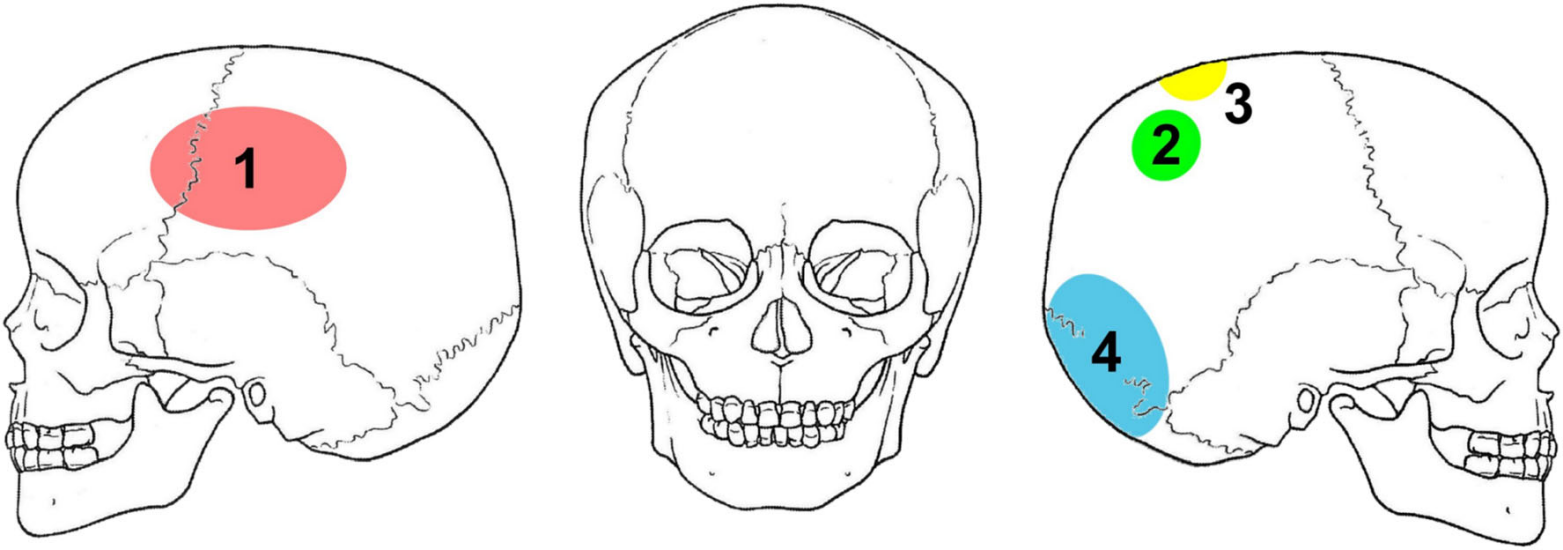
Schematic representation of the lesions at the skull of the 5–6-year-old child from Pit 1981/Grube 3

Most striking are the four perimortal blunt-force traumas at the cranium (Figs 5 and 6). One (Fig 6: 1) is an antero-posteriorly oriented, oval impression fracture measuring 60 × 35 mm with partial penetration of the inner and outer table located at the left side of the frontal and parietal bone in the area of the coronal suture. Two smaller (Fig. 6, 2–3: 25 × 25 mm), round to oval comminuted lesions with linear and radial fracture lines penetrated the right parietal bone in the area of parietal tubercle and next to the sagittal suture. The fourth large (Fig. 6, 4: 45 × 45 mm), circular depression fracture is located at the right parietal/occipital bone in the area of lambdoid suture and completely penetrated the skull.

Perimortal fractures, by definition, occur around the time of death (Cattaneo and Cappella 2017). It cannot be determined with certainty whether the individual was alive or dead when blows affected the bone, but it can be determined that: (1) no signs of healing are present that would suggest the individual survived and (2) the bone was fresh and still contained enough collagen and elastin to cause specific breakage patterns, which would not be present after the decomposition of these elements.

The blows to the head came from several directions and would have been deadly. The first lesion at the left side of the frontal and parietal bone is in a typical location for a right-handed perpetrator facing the victim; it is located above the hat brim line, which is used in forensic science to differentiate blows from falls (Kremer et al. 2008; Kremer and Sauvageau 2009; Spannagl-Steiner et al. 2016). The large lesion at the right parietal/occipital bone below the hat brim line, in contrast, might have resulted from a subsequent fall on the back of the head. A large, blunt weapon or tool was used as a murder weapon; possible objects include the cylindrical loom weight found with the child, a stone, wooden club or the blunt end of a shafted bronze axe. The two smaller lesions (2, 3) at the right parietal bone were likely inflicted by a different weapon with a smaller diameter.

## Methods

### Sex identification via peptides in dental enamel

Morphological sex determination of juveniles is notoriously difficult, and although genetic sexing has recently emerged as an alternative, its application is limited by the high costs and limited preservation of nuclear DNA. In recent years, however, a new method of sex determination via sexually dimorphic amelogenin protein fragments in human tooth enamel has opened up new avenues (Parker et al. 2019; Stewart et al. 2016, 2017). The method is minimally destructive, as only a small amount of enamel is needed for the analysis.

Tooth enamel contains sex chromosome-linked isoforms of amelogenin, an enamel forming protein, which preserve well even in archaeological specimens. The shared amino acid sequence identity of 88% between AMELX isoform 1 (Q99217-1) and AMELY isoform 1 (Q99218-1) allows a distinct interpretation of unique peptides originating from either isoform.

For the Bronze Age child from Schleinbach, the first and second deciduous molars remained in the dental alveoli of both the mandible and the maxilla; in addition, the upper right deciduous first incisor (FDI 51) and the lower left deciduous canine (FDI 73) are present in isolation. We used the lower left deciduous canine for the analysis and mechanically cleaned a 2.5 × 2.5 mm area at the outer surface of the tooth.

The samples were prepared using a slightly modified version of the previously described protocol by Stewart et al. (2017). In order to avoid potential sample cross-contamination, samples were prepared in an isolated fashion on workbenches not dedicated to proteomics sample preparation (Hendy et al. 2018). A small fraction of the tooth enamel was abraded using fine grit sandpaper. The tooth was subsequently washed with 4% (*v*/*v*) hydrogen peroxide (8070.1; Carl Roth) and rinsed with MS-grade water (83645.320; VWR). The abraded part of the tooth’s surface was immersed in 120 μL 5% (*v*/*v*) hydrochloric acid (1.00317.100; Merck) placed in the cap of a 0.5-mL Eppendorf microcentrifuge tube and etched for 2 min; the first etch solution was discarded. A second etch was performed similarly to the first one, which was further processed. C18 ZipTip (87782; Pierce® C18 Tips, Thermo Scientific) were used for the peptide clean-up procedure. The C18 ZipTip conditioning was performed by pipetting 3 times 10 μL 100% acetonitrile (83639.320; VWR), followed by 3 times 10 μL 0.1% (*v*/*v*) formic acid (84865.180; VWR). Each draw was discarded. The etch solution was bound to the resin by pipetting the etch solution into a clean microcentrifuge tube, followed by pipetting up and down in the transferred solution until total pipetting steps were equal to 20 times pipetting. The resin was washed by pipetting 10 μL 0.1% formic acid 6 times; each draw was discarded. Elution of peptides was performed by pipetting 2 times 10 μL each elution buffer (60% ACN, 0.1% FA) into a clean 1.5 mL Eppendorf microcentrifuge tube. The sample was dried in a vacuum concentrator and reconstituted in 2 μL 30% formic acid solution containing four synthetic standard peptides (10 fM, Supplementary Table S1); for internal quality control, 10 μL eluent A (97.9% MS grade water, 2% MS grade ACN, 0.1% FA) was added.

Analysis was performed employing a Dionex Ultimate 3000 nanoLC-system coupled to a Q Exactive classic orbitrap mass spectrometer equipped with a nanospray ion source. Measurement parameters for LC as well as MS conditions were adopted based on a recently published method (Stewart et al. 2017). In short, peptide trapping was performed with a Thermo Scientific Acclaim™ PepMap™ 100 C18 column with 20 mm length (100 μM ID, 5 μM particle size), followed by peptide separation using a Thermo Scientific Acclaim™ PepMap™ 100 C18 column with 500 mm length (75 μM ID, 3 μM particle size). A nonlinear gradient from 1 to 45% eluent B (20% MS grade water, 79.9% MS grade ACN, 0.1% FA) over 55 min at a flow rate of 300 nL/min with subsequent increase to 99% eluent B for 5 min followed by column equilibration at 1% eluent B for 19 min. The mass spectrometer was set to a scan range from 300 to 1400 *m*/*z* at MS1 level. Resolution was set to 70,000 and 17,500 at 200 *m*/*z* with a maximum IT of 50 and 150 ms and AGC target of 3e6 and 2e4 for MS1 and MS2 scans, respectively. For MS2 scans, an isolation width of 1.8 *m*/*z* was chosen and only charge states from 2 to 4 were allowed for HCD fragmentation at 30% normalised collision energy.

Automated data interpretation was carried out with the MaxQuant software (version 1.6.3.4) employing the implemented Andromeda search engine (Cox and Mann 2008). For positive protein identification, at least one unique peptide had to be detected. Peptide mass tolerance at MS1 level was set to 50 and 25 ppm for the first and the main search, respectively. Fragment mass tolerance was set to 20 ppm. The false discovery rate (FDR) was set to 0.01 both on peptide and protein level. ‘Match between runs’ was enabled and the alignment and match time window set to 15 and 1 min, respectively. The database applied for protein identification was the human Uniprot database (version 03/2018, with 20316 reviewed entries). Methionine oxidation and N-terminal acetylation were set as variable modifications. Obtained proteomic results on peptide and protein level can be found in Supplementary Table S2.

Furthermore, a MS1 filtering step via the software Skyline (version 20.1.0.79; Schilling et al. 2012) was implemented to monitor high quality peptide candidates, obtained from the abovementioned MaxQuant search, in respect to their mass deviation and isotope distribution proportions. Raw data was imported and extracted ion chromatograms for two peptide candidates, each representing a unique peptide containing the target sequence for both sex-related isoforms, with a MaxQuant score of at least 40 were created. Isotope distribution proportions were monitored via the Skyline idotp score, with an applied cut-off of > 0.95 and a mass tolerance of 5 ppm.

## Results

**Fig. 7:**
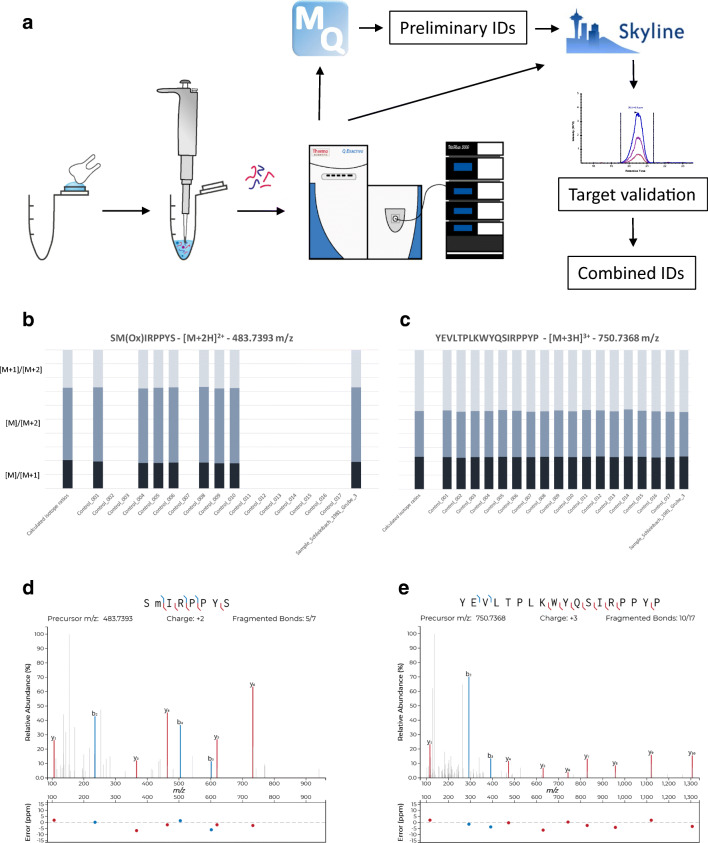
**a** Schematic overview of sample preparation and measurement workflow adapted from Stewart et al. (2017) with extended data evaluation scheme. **b** Comparison of isotope ratios of peptide SM(Ox)IRPPYS between M and M+1 (dark blue), M and M+2 (light blue), and M+1 and M+2. Theoretical calculated isotope ratios are given on the left-hand side as reference. **c** Comparison of isotope ratios of peptide YEVLTPLKWYQSIRPPYP between M and M+1 (dark blue), M and M+2 (light blue), and M+1 and M+2. Theoretical calculated isotope ratios are given on the left-hand side as reference. **d** MS/MS fragment spectrum of precursor mentioned in Fig. 7b from sample SB with annotated b and y chain matches and corresponding mass errors in ppm (Brademan et al. 2019). **e** MS/MS fragment spectrum of precursor mentioned in Fig. 7c from sample SB with annotated b and y chain matches and corresponding mass errors in ppm (Brademan et al. 2019)

The combination of multiple proteomic analysis strategies, empowered by algorithms with unbiased statistical data evaluation and comprehensive manual curation of peptide signals with rigorous filtering of mass tolerance and isotope distribution proportions allows a high confidence sex estimation (Fig. 7a).

A control cohort of 17 modern deciduous teeth, consisting of seven male and ten female individuals, was employed. In total, the analysis yielded 286 peptides corresponding to 39 proteins, among them typical enamel-derived proteins such as ameloblastin (AMBN), enamelin (ENAM) or amelogenin (AMELX/AMELY) (Supplementary Table S2). From this list, high scoring peptides containing the target sequence of both sex-related isoforms were chosen for detailed signal extraction. The peptides SM(Ox)IRPPYS ([M+2H]^2+^ = 483.7393 *m*/*z*) and YEVLTPLKWYQSIRPPYP ([M+3H]^3+^ = 750.7368 *m*/*z*) were chosen as diagnostic peptides with a MaxQuant ID score of 77.7 and 130.0, mean idotp score of 0.9825 and 0.9983 and mass tolerance of 5 ppm, respectively.

A male origin of the sample is indicated by detection of both diagnostic peptides derived from each isoform (Fig. 7b, c). As an additional verification, MS/MS spectra of both peptides measured in the sample of the Schleinbach child are illustrated in Fig. 7d, e.

Additionally, as shown in Table 1, we employed an automated and unbiased data analysis via the MaxQuant software (Cox and Mann 2008). With this approach, the identification of both amelogenin isoforms with a FDR ≤ 0.01 on protein, as well as on peptide level, was possible. Label-free quantification (LFQ) values indicate detected protein intensities from corresponding peptide signals, strengthening the chosen data analysis strategy with the unambiguous assignment of detected AMELY signals only for the seven male controls and the sample of the Schleinbach child (Supplementary Fig. S1).

**Table 1:** AMELX/AMELY protein identification and quantification results obtained via MaxQuant software

			Protein isoform	
			AMELY	AMELX
		Uniprot Accession ID	Q99218-1	Q99217
		Unique sequence coverage (%)	10.9	19.4
		MS/MS count	35	513
		Protein identification score	41.075	323.31
Samples	Known sex	Predicted sex	LFQ Intensity	
Control_001	Male	Male	28.4338	32.6342
Control_002	Female	Female	NaN	33.0124
Control_003	Female	Female	NaN	32.691
Control_004	Male	Male	27.9284	32.4267
Control_005	Male	Male	28.6873	32.8285
Control_006	Male	Male	28.6071	32.7046
Control_007	Female	Female	NaN	32.7211
Control_008	Male	Male	29.0894	32.4398
Control_009	Male	Male	28.7453	32.2737
Control_010	Male	Male	27.9927	32.841
Control_011	Female	Female	NaN	32.4098
Control_012	Female	Female	NaN	32.4978
Control_013	Female	Female	NaN	32.7599
Control_014	Female	Female	NaN	30.7015
Control_015	Female	Female	NaN	33.1395
Control_016	Female	Female	NaN	33.0391
Control_017	Female	Female	NaN	33.6924
Schleinbach_1981_Grube_3	NaN	Male	28.949	32.9739

Mass spectrometry data is publicly accessible via the PRIDE archive (Perez-Riverol et al. 2019) under the accession PXD018069 (http://www.proteomexchange.org). In addition to data on sex, the shotgun LC-MS/MS method delivers data on other proteins that may later be analysed in the light of other questions, such as diseases (Supplementary Table S2).

**Fig. 8:**
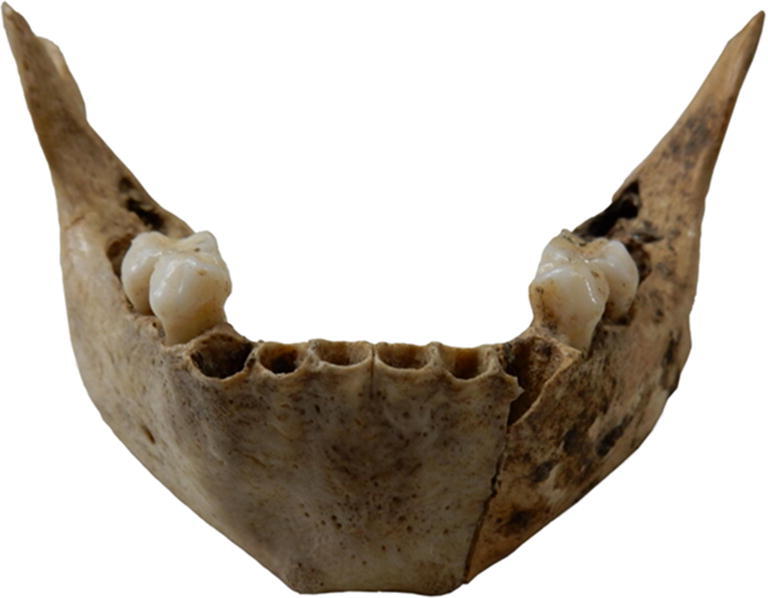
Mandible of the 5–6-year-old child from Pit 1981/Grube 3

The morphology of the child’s mandible (Fig. 8) aligns with the criteria for male sex identification, for which an accuracy between 69 and 90% has been reported. As typical for male juveniles, the ‘straight sides of the corpus diverge angle sharply in the canine region and form a \_/-shaped body’ (Loth and Henneberg 2001). The chin region is generally more prominent in boys, and the protrusion of the chin region is wide and angular (Schutkowski 1993).

## Discussion

### The context of violence

In the Bronze Age, children were often casualties of conflict between communities. Archaeological evidence of warfare in the form of weaponry, defensive architecture and trauma on human remains is omnipresent in the Bronze Age (e.g. Osgood and Monks 2000; Otto et al. 2006; Parker Pearson and Thorpe 2005; Peter-Röcher 2007; Uckelmann and Mödlinger 2011). Bronze Age warfare has been described as war-bands engaging in inter-group raiding (Harding 2007), but it may also have a religious and ritual role (Harrison 2004; Kristiansen and Larsson 2005). Male identity appears strongly connected to the warrior identity, which includes ideas of honour, prestige and bodily beauty alongside violence (Rebay-Salisbury 2017; Treherne 1995). The killing of male children in particular targeted the male linage, whereas female children were more likely to be captured and integrated in the victorious societies in some way (Cameron 2016).

At Schleinbach, seven of 37 (18.9 %) observable individuals had cranial injuries, primarily without healing signs. Two male individuals aged 27–30 and 30–35 years and buried together in close bodily contact in one grave most likely died of nearly identical blunt force traumas at the frontal section of their left parietal bones. The burial of an adult male with three children aged 3–4, 7–9 and 12 years at death in a pit, in contrast, was much less formal. A perimortal burst fracture was discovered at the left parietal bone of the youngest child, but it seems likely that the other individuals were also killed in the same violent event (Rebay-Salisbury 2018). Similar findings in the region include the burial from Herzogbirbaum, where a group of three women, an adolescent and two children were found in a pit, four of them with perimortal fractures (Lauermann and Pany-Kucera 2012). Conflicts between neighbouring villages or groups appear the most likely explanations for these findings.

Some evidence, however, point to inter-personal violence within the group rather than between-group conflicts. Multiple fractures, for example, are frequently paired with general indicators of poor health, suggesting injury recidivism as an indicator for systematic mistreatment and marginalisation within the community (Martin et al. 2010, 2012). The fact that cemetery demographics frequently point to the underrepresentation of children under one (Beilke-Vogt 2004) suggests they were not considered persons in the same way as older children and adults.

### Killing children

The killing of children by their parents is terminologically classified by the children’s age, from neonaticide, which refers to killing within the first day or week of life, to infanticide, the killing of under 1-year olds, and filicide, the killing of a child of any age by their parent (Friedman et al. 2005). Infanticide, the killing of unwanted babies, can be considered a global, cross-cultural phenomenon if effective birth control is absent and resources are limited (Gammeltoft and Wahlberg 2014; Mays 2000). The most credible evidence of ritual child sacrifice has been documented in Ancient Carthage (Xella et al. 2013), in Archaic societies of the Ohio Valley (Claassen 2013) and the Inka Empire (Blom 2018), with many more ambiguous and hotly-debated cases from all over the world. Infants may be actively killed, but it is difficult enough to keep a new-born alive; exposure and neglect are therefore equally suited methods of infanticide (cf. Scheper-Hughes 1992).

As an institutionalised and legal form of killing children, infanticide nevertheless usually follows social rules and restrictions. In Ancient Rome, for example, infanticide was legal if the child was sickly or disabled. Sex and birth order also play a role: all sons and the first-born-daughter had to be raised (Krauße 1998). Many cultures practice sex-biased infanticide, which is why it is crucial to determine the sex of slain children in archaeological populations. In patriarchal societies, in which the transfer of wealth between the generations and the care for the elderly is governed by gender-based rules, girls are often the victims (Meyer et al. 2001), although female infanticide is not a universal phenomenon (Scott 2001). A striking example of male-biased infanticide emerged from the genetic sex determination of 100 neonates discovered in a sewer beneath a Roman bathhouse in Ashkelon, Israel. Fourteen of the 19 neonates for which DNA extraction was successful were boys, which suggests that the girls might have been raised to work in the brothel, whereas the boys were disposed of (Faerman et al. 1998). It is unclear, however, how representative this extraordinary context is; no sex bias was found in the infants buried in a settlement context of Romano-British Hambleden (Abu-Mandil Hassan et al. 2014).

The Early Bronze Age cemetery at Mokrin, Serbia (Rega 1997; Scott 2001), dating from c. 2100 to 1500 bc and thus roughly contemporaneous to our case from Schleinbach, is characterised by the absence of children under one, and a statistically significant excess of female children between 1 and 6 years. Elizabeth Rega concludes that this might point to intentional killing or neglect of male neonates, resulting in a larger proportion of female children in the population reflected in the cemetery demography (Rega 1997). This is, however, not as straightforward as presented. At this cemetery, adults were buried according to gender-based rules that dictate the position of the body in the grave. Females were placed in flexed position on the right side, with the head in the south; males were buried on the left side, head north. It is assumed that children follow the same rules, and for older children, this has been confirmed by a sex assessment of permanent teeth (Rega 1997). Younger children, who may not yet have been ascribed a specific gender by society, in contrast, may have been placed as girls because they still belong their mother’s sphere of gender, similar to young boys in early modern society before they were switched from gowns to trousers (Ashelford 2011). Sex-determining region Y (SRY) and amelogenin DNA analysis of children’s remains from the Bell-Beaker cemetery of Hoštice, Czechia (Turek 2014; Vaňharová 2011), have shown that most buried children were males, even if they were buried according to female (gendered) body positioning rules.

Reviews and statistical analysis of forensic cases (Dawson 2015; Mariano et al. 2014) of child murder provide interesting insights into age and gender, methods and motives of killing children. In modern, primarily western societies, 61% of child murders under the age of five were committed by parents (Friedman et al. 2005). The murdered children were girls and boys, with no significant bias. Mother’s victims were usually younger than father’s (Putkonen et al. 2011); while neonaticides and infanticides were primarily carried out by the mother, fathers were more likely responsible for the murders of children over eight (Greenfeld and Snell 1999). Head trauma has been noted as the most common method used by both female and male murderers (Resnick 1969). In a classic study, Resnick assigned 131 filicides in five distinct groups according to motive: altruistic, acutely psychotic, unwanted child, accidental and spouse revenge (Meyer et al. 2001; Resnick 1969). The altruistic motive includes both cases of attempted suicide by the parents that did not want to abandon their children (38%) and attempts to alleviate real or delusional suffering (11%). Scott (1973) defines ‘mercy killings’ more narrowly, as cases ‘in which there is a real degree of suffering in the victim and the absence of secondary gain for the parents’.

In the light of the pathologies discovered in the 5-6-year-old boy from Schleinbach, a ‘mercy killing’ cannot be excluded. The child suffered from a painful ear infection but did not have any other traces of violence found in battered children, such as healed or partly healed fractures on the few bones that are available for study. The symptoms might have been accompanied by fever, difficulty sleeping, crying, preventing siblings from sleeping and keeping the entire household awake, which—conversely—might have triggered aggression and violence. If the boy was affected by a hearing impairment, accompanied by a diminished capacity to speak and engage within the community (Daniel et al. 1988), this might have contributed to his weak social position. The assault may have happened in a domestic context, with the loom-weight as a possible murder weapon deposited in the same layer of the pit. The mother—or any other member of the community—might have killed the child out of love, not because of a lack of affection (cf. Eriksen 2017). Not being able to alleviate the pain of the sick child, death might have been the only way to relieve his suffering in a hopeless situation.

## Conclusions

The presented case of a murdered boy found in an Early Bronze Age settlement pit highlights the importance of sex identification of juvenile remains for an in-depth interpretation of infanticide and filicide. The identification of sex-specific peptides in human enamel by nanoflow liquid chromatography-tandem mass spectrometry (nanoLC-MS/MS), applied for the first time in Austria for the present case study, is a highly accurate method of sexing juvenile remains, particularly when multiple proteomic data interpretation strategies are combined. Accurate data on the sex of buried infants and children opens up a range of new research avenues on sex-specific mortality and burial practices, sex-preferences, and demography. A broad, systematic application of proteomics will clarify whether or not sex selection took place after birth, and whether infanticide and filicide affected more girls or boys. We can investigate whether girls and boys were treated equally as babies and small children, for example in terms of access to food or formal burial rites. We will be able to understand if girls and boys were classified according to sex from birth, or later, as they matured and ‘learned’ gender (cf. Sofaer 2006). In summary, we can learn a lot about value systems linked to gender, about power relationships between the sexes, and about how they developed in past societies.

## Electronic supplementary material

ESM 1 (PDF 330 kb)

ESM 2 (XLSX 8 kb)

ESM 3 (XLSX 235 kb)
