# Supplementary figures and images for: Child murder in the Early Bronze Age: proteomic sex identification of a cold case from Schleinbach, Austria
Source: Archaeol Anthropol Sci. 2020 Oct 23;12(11):265. doi: 10.1007/s12520-020-01199-8 (PMC7584537; doi:10.1007/s12520-020-01199-8)

Control\_001

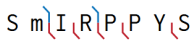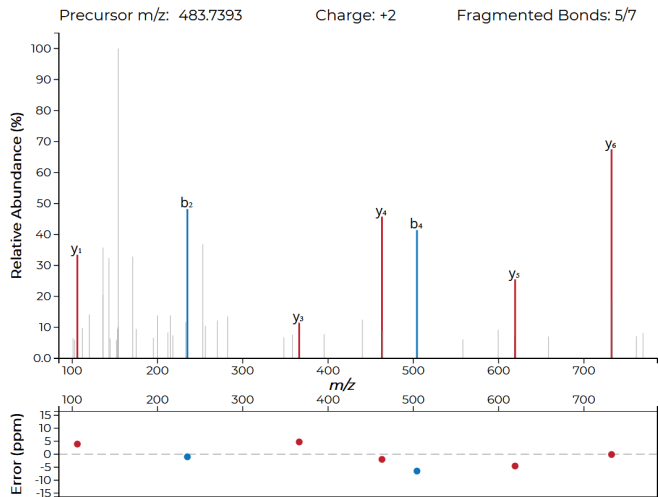

Control\_004

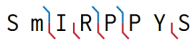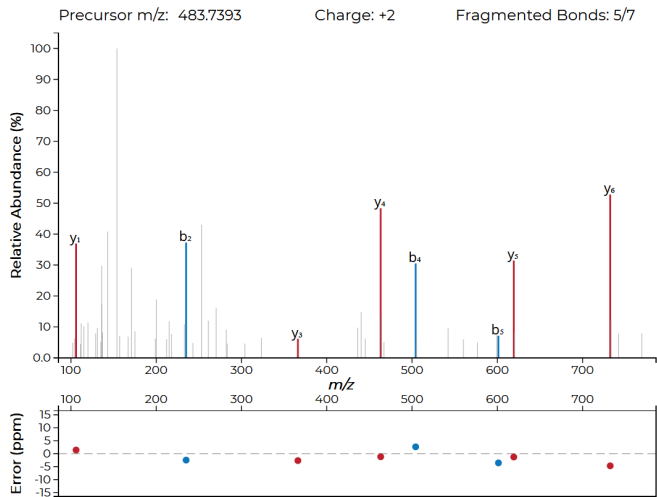

Control\_005

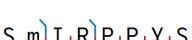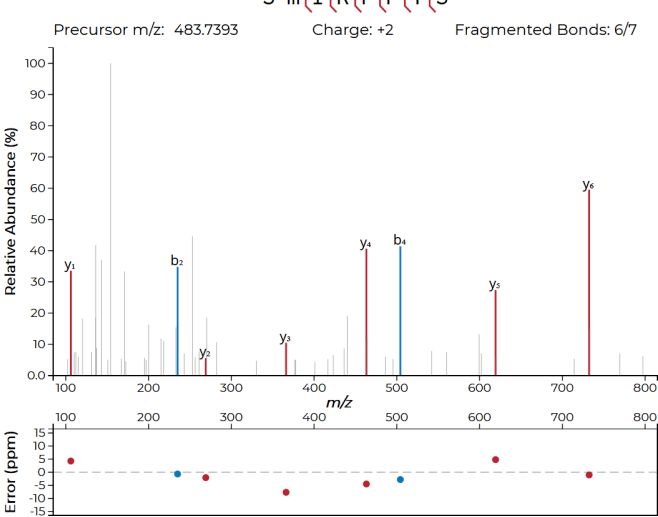

Control\_006

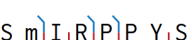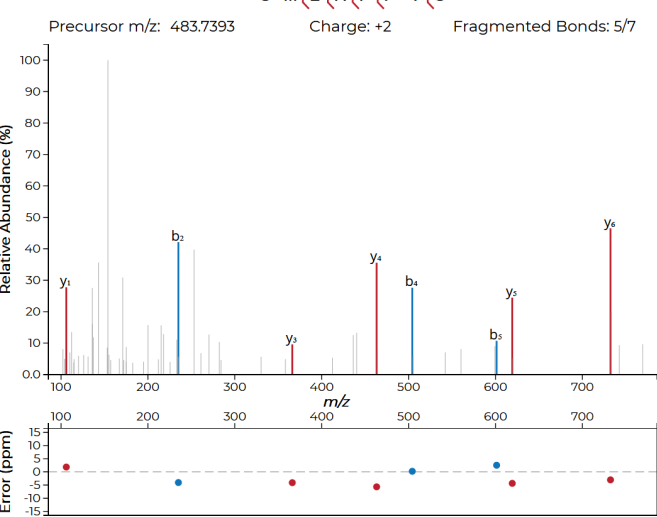

Control\_008

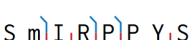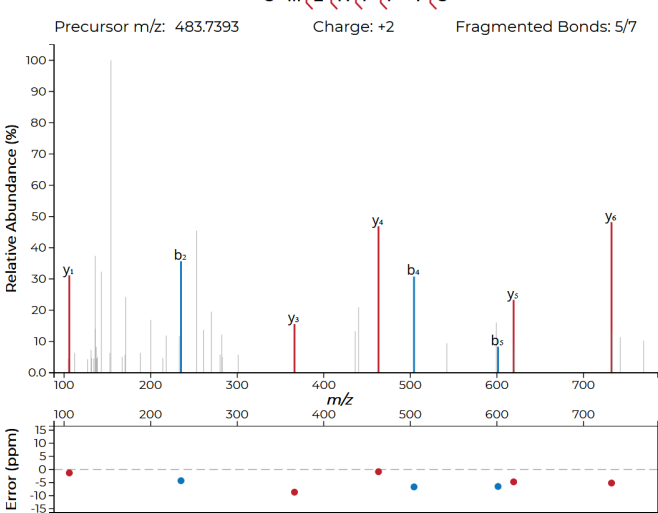

Control\_009

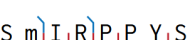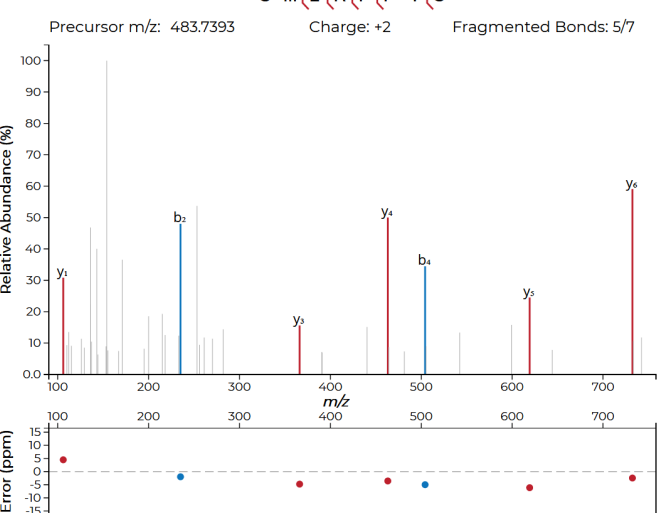

Control\_010

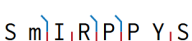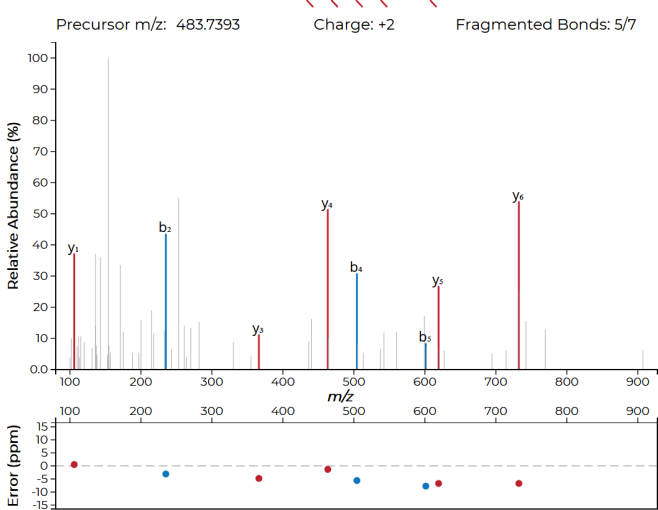

Sample\_SB

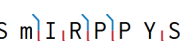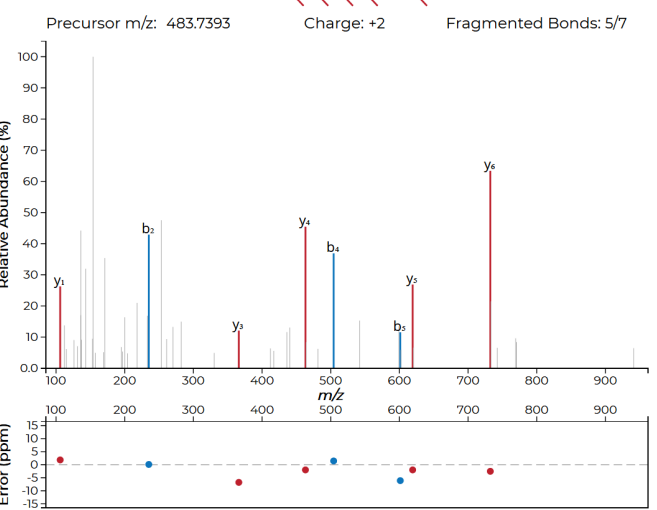

Supplement: Supplementary file 1 — (PDF 330 kb) [file 12520_2020_1199_MOESM1_ESM.pdf]
